# Reoperative pouch surgery for suspected Crohn's‐related complications aided by biologic coverage: Early experience from an inflammatory bowel disease center

**DOI:** 10.1002/ags3.70016

**Published:** 2025-03-25

**Authors:** Mehmet Gulmez, Daniel J. Wong, Ariela K. Holmer, Eren Esen, Shannon Chang, Arman Erkan, David Hudesman, Andre da Luz Moreira, Feza H. Remzi

**Affiliations:** ^1^ Center for Advanced Inflammatory Bowel Disease Care Northwell Health Manhasset New York USA; ^2^ Inflammatory Bowel Disease Center, Department of Surgery, NYU Langone Health New York New York USA; ^3^ Division of Colorectal Surgery, Harvard Medical School Beth Israel Deaconess Medical Center Boston Massachusetts USA; ^4^ Inflammatory Bowel Disease Center, Division of Gastroenterology NYU Langone Health New York New York USA

**Keywords:** biologic, Crohn's disease, pouch, revisional/redo IPAA

## Abstract

**Aim:**

In patients with failing ileo‐anal pouches there is often diagnostic uncertainty. In this setting, we may offer revisional pouch surgery with biologic “coverage” for presumed Crohn's disease (CD) which enables an alternative to pouch excision and end ileostomy to highly motivated patients. The aim of this study is to assess postoperative outcomes in patients who underwent revisional/redo ileal pouch anal anastomosis (IPAA) for failing pouches with biologic coverage for possible CD.

**Methods:**

This is a retrospective cross‐sectional study based on data from a tertiary inflammatory bowel disease center. Patients who underwent revisional/redo IPAA surgery between September 2016 and December 2022 were included. The primary outcome measure was the rate of functioning pouch.

**Results:**

Of the 213 patients who underwent revisional/redo IPAA surgery, 17 underwent redo IPAA surgery with biologic coverage due to concern for CD. An additional seven patients were started on biologics between the two operative stages of redo IPAA surgery. At a median follow‐up of 17 months, the functioning pouch rate was 75%.

**Conclusions:**

Revisional IPAA surgery for suspected CD‐related complications leading to pouch failure, in conjunction with concurrent medical therapy, provides a stoma‐free alternative to patients otherwise facing pouch excision and end ileostomy. Despite the limited number of patients and varying follow‐up times, this approach shows promise for maintaining pouch function in a challenging patient population.

## Graphical abstract

This paper demonstrates that revisional/redo ileal pouch anal anastomosis (IPAA) surgery with concurrent biologic therapy provides a viable, stoma‐free alternative for patients with Crohn's disease‐like complications, showing a high functioning pouch rate and expanding options for managing pouch failure.

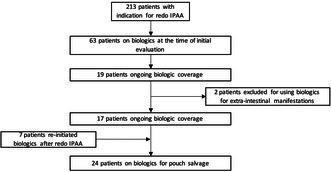

## 1 INTRODUCTION

Ileal pouch‐anal anastomosis (IPAA) is the preferred surgical option to restore intestinal continuity after proctocolectomy in patients with ulcerative colitis (UC), selected patients with colonic Crohn's disease (CD), and familial adenomatous polyposis (FAP).^1^ The functional outcomes and improvement in quality of life (QoL) following IPAA are well‐established. However favorable results can be compromised in patients with postoperative complications such as pouch obstruction, chronic pouchitis, or fistulous disease of the pouch.^2^ Untreated complications may lead to pouch failure, necessitating pouch resection, diversion, or revisional pouch surgery which occurs in as many as 15% of patients.^3^

Pouch failure can be salvaged through revisional/redo IPAA surgery.^4^ The success of revisional/redo IPAA surgery depends on identifying and correcting the underlying cause of the failed pouch. Patients presenting with suspected CD‐related complications of the pouch can be particularly challenging to treat. Classically, CD‐related complications of the pouch manifest with fistulas, abscesses, pouch inlet or afferent limb strictures, or refractory pouch inflammation.^5^ Patients with intestinal or perianal CD are traditionally advised against salvage surgery due to the high rates of complications and subsequent pouch failure associated with CD as compared to ulcerative colitis or indeterminate colitis (IC).^6^

However, in our experience, failing pouches are often labeled as having CD when the etiology of pouch failure may be mechanical or technical. These complications may result in chronic inflammation or persistent fistulae which can be indistinguishable from CD of the pouch. For example, pouch‐related fistulas that develop in the setting of an anastomotic or tip of the J leak are often a surgical rather than a CD‐related complication. Pouch outlet issues such as a long rectal cuff, pouch constriction from residual mesorectum, or anastomotic strictures lead to obstruction and stasis causing chronic inflammation which can be mistaken as CD. Mechanical issues such as a twisting of the mesentery or pouch elongation from outlet obstruction may appear as inlet issues. Given the diagnostic uncertainty in some patients with a failing pouch, we have offered revisional pouch surgery in the setting of biologic “coverage” for presumed CD for a select population of highly motivated patients with a severe aversion to a permanent ileostomy. Herein, we share our experience with patients who underwent salvage medical therapy after revisional/redo IPAA with suspicion for CD.

## 2 METHODS

Following institutional review board approval (i23‐01115) of a prospectively maintained pouch registry, a retrospective analysis was conducted of all patients undergoing revisional pouch surgery between September 2016 and December 2022 at the Inflammatory Bowel Disease (IBD) Center of NYU Langone Hospital. Patient characteristics, operative details, and postoperative short‐ and long‐term outcomes were abstracted from the medical record. We also determined functional results using the Cleveland Clinic Foundation (CCF) Pelvic Pouch Questionnaire which included details pertaining to bowel frequency (number of bowel movements per 24 h), urgency (inability to defer bowel movements for more than 15 min), fecal incontinence (inadvertent passage of liquid or solid stool), need to wear pads, and restrictions (dietary, social, work, sexual). The Cleveland Global Quality of Life (CGQL) score was used to perform assessment of quality of life.^7^ Patients were asked to rate current quality of life, current quality of health and current energy level, each on a scale of 0 to 10 (0, worst; 10, best). The scores were added, and the final CGQL utility score was obtained by dividing the sum by 30.

All patients presenting for revisional pouch surgery underwent a comprehensive evaluation including: review of prior operative and pathology records; examination under anesthesia (EUA) and pouchoscopy; water‐soluble contrast enema (WSCE); and magnetic resonance imaging (MRI) of the pelvis. Fistulas were classified using the findings of the preoperative workup as well as intraoperative findings at the time of the revisional pouch surgery. Revisional pouch surgery is conducted in three stages: (1) creation of a diverting ileostomy if not already performed, (2) a redo operation with a diverting stoma, and (3) ileostomy reversal.

Following multidisciplinary evaluation, highly motivated patients in whom CD was a diagnostic possibility and who were deemed suitable candidates for revisional/redo IPAA were operated on with biologic “coverage” for CD. Biologic coverage may have been initiated before initial evaluation for revisional/redo IPAA surgery or following evaluation during the stages of revisional/redo IPAA surgery. This decision was individualized for each patient with a multidisciplinary approach and included expertise from a gastroenterologist who specializes in IBD. CD‐like features of the pouch were defined using the most widely recognized diagnostic criteria for this condition. These criteria include the presence of one or more fistulae originating from the pouch or afferent limb, the development of strictures or stenoses in the pouch body, pouch inlet, or afferent limb, and/or the presence of pre‐pouch ileitis. Fistulas were classified using the findings of the preoperative workup as well as intraoperative findings at the time of the revisional pouch surgery. The timing of fistula development is a critical factor, as fistulae that develop more than 12 months after IPAA surgery are more indicative of CD of the pouch rather than a postoperative complication. At the same time, we understand that a definitive diagnosis of CD is commonly impossible to achieve with absolute certainty. Biologics or small molecules initiated included the following therapies: anti‐tumor necrosis factor (TNF) (infliximab, adalimumab, or certolizumab pegol), anti‐integrin (vedolizumab), anti‐interleukin 12/23 (ustekinumab), and janus kinase inhibitor (tofacitinib) during redo IPAA surgery. History of previous biologic (or small molecule) use, indications, type, and the initiation time of the medical therapy were collected. Any change in the medical therapy during the perioperative period was also documented.

### 2.1 Statistical analysis

Categorical variables were given as frequency (*n*) and numeric variables were given as mean ± standard deviation (SD) or median (interquartile range). IBM SPSS Statistics Version 28.0 (IBM Corp., Armonk, NY) was used for this analysis.

## 3 RESULTS

**FIGURE 1:**
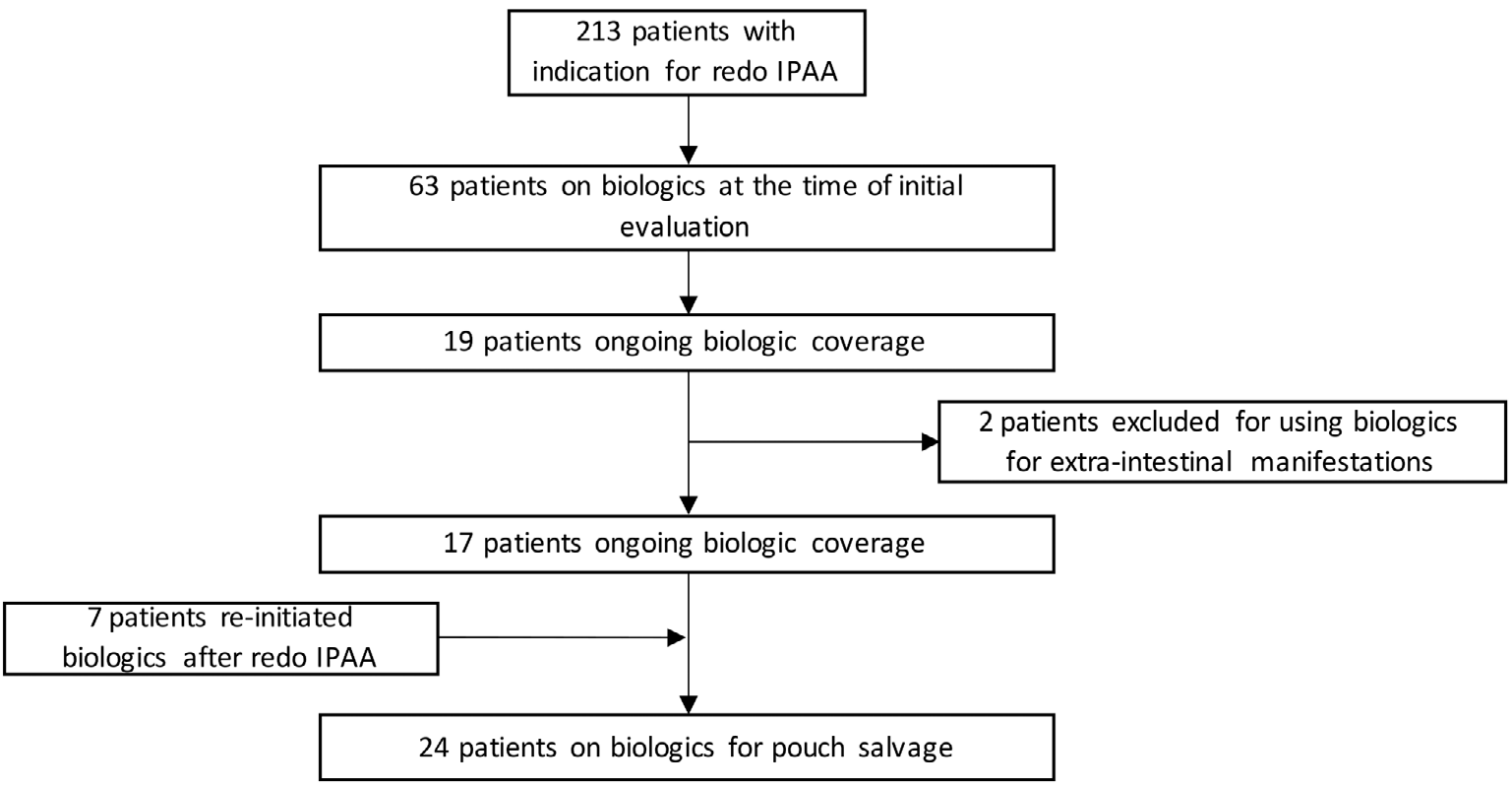
Flowchart detailing patient selection for the study.

A total of 213 patients who experienced pouch failure underwent revisional/redo IPAA surgery. Among them, 63 patients were found to be on biologic therapy within 3 months before their initial evaluation at our center. Ultimately, upon initial evaluation 19 of these 63 patients underwent redo IPAA surgery with ongoing biologic coverage. However, two of these 19 patients were excluded for using biologics for extra‐intestinal manifestations of IBD. During redo IPAA surgery, in seven patients who had a history of biologic use, biologics were re‐initiated before the final stage of redo IPAA surgery, resulting in a total of 24 patients received biologic coverage for pouch salvage in the setting of suspected CD (Figure 1).

Table 1 includes patient demographics and characteristics of the primary and redo pouch surgery. The median age was 40 (29–46) years, and 14 (58%) were female. Median time between primary and redo pouch surgery was 5 years.^3^, ^4^, ^5^, ^6^, ^7^, ^8^, ^9^, ^10^, ^11^, ^12^ All patients had diverting loop ileostomy before redo pouch surgery for at least 6 months. Eighteen patients (75%) presented with pelvic sepsis and 12 patients presented with one or more fistulae, and of these 12 patients, the origin of the fistula was identified at the previous pouch anal anastomosis site in 11 patients. Pouch excision with new pouch construction was performed in 11 out of 24 patients (46%), and all new pouches were created in the J pouch configuration. The majority of patients underwent hand‐sewn anastomosis and mucosectomy (73%).

**TABLE 1:** Patient and primary/redo pouch characteristics, *N* = 24.

Variables	*n* (%)
Demographic characteristics	
Age, years	40 (29–46)
Female	14 (58%)
BMI (kg/m^2^)	22 (20–25)
Smoking status	
Never	18 (75%)
Former	5 (21%)
Current	1 (4%)
ASA score	
2	17 (71%)
3	7 (29%)
Clinical characteristics of the primary pouch surgery	
Stages for primary pouch	
Three stage	19 (79%)
Two stage	3 (13%)
One stage	2 (8%)
Approach type of the primary pouch	
Open	14 (58%)
Laparoscopic	10 (42%)
Configuration type of the primary pouch	
J pouch	24 (100%)
Clinical Characteristics of the redo pouch surgery	
Time between primary and redo pouch, years	5 (3–12)
Indications for redo pouch^a^	
Pelvic sepsis	18 (75%)
Fistula	12 (50%)
Origin of fistula (*n* = 12)	
IPAA	11/12 (92%)
Tip of J	1/12 (8%)
Type of fistula (*n* = 12)	
Pouch‐vaginal	6/12 (50%)
Perianal	5/12 (42%)
Both	1/12
Seton (previously placed)	4 (17%)
Mechanical	17 (71%)
Long cuff and remnant mesorectum	9 (38%)
Twist	7 (29%)
Stricture	
Anastomosis	8 (33%)
Pre‐pouch inlet	3 (12%)
Small pouch	4 (17%)
Type of the redo pouch	
Redo IPAA with old pouch	11 (46%)
Redo IPAA with revision	5/11 (45%)
Redo IPAA without revision	6/11 (55%)
Redo IPAA with new pouch	11 (46%)
Pouch revision alone (Tip of J resection)	2 (8%)
Configuration type of the redo pouch	
J pouch	24 (100%)
Anastomosis type (*n* = 22)	
Stapled	6 (27%)
Handsewn	16 (73%)
Indication for biologic coverage (*n* = 24)	
Ongoing biologic coverage (*n* = 17)	
CD like features of the pouch	7 (29%)
Perianal fistulizing disease	6 (25%)
Afferent limb stricture	4 (17%)
Re‐initiated biologic coverage^b^ (*n* = 7)	
De novo fistula development	3 (13%)
Anal canal ulcers	2 (8%)
Pouch body sinus	1 (4%)
Pathology from pouch excision showing concerning for CD	1 (4%)
Type of biologic	
Ustekinumab	10 (42%)
Vedolizumab	4 (17%)
Adalimumab	4 (17%)
Infliximab	3 (12%)
Tofacitinib	3 (12%)

^a^ Several patients had several indications.

^b^ Re‐initiated between second and third stages of surgery.

Indications and type of biologic coverage are outlined in Table 1. Seven patients (29%) were re‐initiated on biologic therapy after the second stage of revisional/redo IPAA. The main reasons for re‐initiation of biologic therapy were de‐novo fistula development, anal canal ulcers, pouch body sinus. One patient re‐initiated biologic therapy according to a pathology report from pouch excision showing transmural inflammation concerning for CD, despite having no signs of active CD. In our series, the majority of patients received ustekinumab (*n* = 10), followed by vedolizumab (*n* = 4), adalimumab (*n* = 4), infliximab (*n* = 3), and tofacitinib (*n* = 3). During follow‐up, six patients underwent a treatment change to an alternative biologic due to the development of a new fistula, unresponsiveness to therapy or presence of antibodies. In two patients, preoperative infliximab was changed to ustekinumab or adalimumab due to a newly diagnosed perianal and pouch vaginal fistula before closure of ileostomy.

In terms of 30‐day and beyond 30‐day surgical outcomes, one patient required a return to the operating room for drainage and vacuum‐assisted closure (VAC) for abdominal wall abscess. Nineteen patients (79.1%) underwent successful ileostomy closure after assessing the redo pouches for reversal of ileostomy with MRI of the pelvis and gastrografin enema. Five patients did not undergo ileostomy closure due to either a pouch‐vaginal fistula (*n* = 1), tip of the J leak requiring re‐redo IPAA (*n* = 1), IPAA leak (*n* = 1), sinus tract being treated with sinusotomy (*n* = 1), or patient preference (*n* = 1). Two patients developed perianal fistulas after ileostomy closure and were managed by a diverting loop ileostomy. Furthermore, following fistula healing, one patient underwent ileostomy reversal and the other patient is still awaiting closure. One patient reported passing air through her vagina, however, no definite fistula was detected upon examination, and her symptoms improved during the follow‐up period without the need for ileostomy creation. Two patients underwent a re‐redo IPAA, including one patient who went directly to a re‐redo IPAA before ileostomy closure for tip of the J leak and one patient who had pouch dysfunction due to septic complications after redo IPAA. Both of the patients had continued biologic therapy during the stages of re‐redo IPAA and secondary revision was successful in one patient. The other patient still waiting for ileostomy closure. In summary, at a median follow‐up of 17 months, 18 (75%) patients had a functioning pouch and no stoma.

Regarding patients' satisfaction with the results of the redo IPAA, eight (44%) patients who had functioning pouch responded to the questionnaire. The median number of bowel movements was six per day. The Cleveland Global Quality of Life score was 8.3,^8^, ^9^, ^10^ and patients were happy with the results. All patients would undergo surgery again if needed and would recommend surgery to other patients (Table 2).

**TABLE 2:** Redo pouch surgery short‐ and long‐term outcomes, *N* = 24.

Operative and postoperative 30‐day outcomes after redo pouch surgery	
Operation time, min	275 (236–305)
Blood loss, cc	250 (200–500)
Length of stay, day	8 (6–10)
Readmission (30‐day)	6 (25%)
Reoperation (30‐day)	1 (4%)
Morbidity (30‐day)	14 (58%)
Type of complication^a^	
Pelvic collection/abscess required drainage	2 (8%)
Leak/fistula	0 (0%)
Wound infection/dehiscence	8 (33%)
Transfusion	5 (13%)
AKI/Dehydration	5 (13%)
SBO/Ileus	2 (8%)
Thrombosis (DVT, PE)	1 (4%)
Urinary retention	1 (4%)
Urinary tract infection	0 (0%)
Cardiopulmonary	0 (0%)
Beyond 30‐day outcomes after redo pouch surgery	
Median follow‐up	17 (10–31)
Beyond 30‐day complications after redo IPAA	
Septic complications	7 (29%)
Pouch dysfunction – underwent re‐redo IPAA, successful	1 (4%)
Stricture	1 (4%)
Ileostomy reversal	19 (79%)
Complications after ileostomy closure	
Perianal fistula, re‐diversion, and reversed again, successful	1/19
Perianal fistula, re‐diversion, pending for closure	1/19
Ileostomy never closed (*n* = 5)	
Tip of J leak ‐ underwent re‐redo IPAA, pending for closure	1/5
Persistent pouch‐vaginal fistula	1/5
Sinus tract treated with sinusotomy	1/5
Anastomotic leak	1/5
Patient preference	1/5
Functioning pouch	18 (75%)
Functional results and Quality of Life scores (*n* = 8)	
Bowel movements	
Daytime	6 (5–7)
Nighttime	1 (0–1)
Total	7 (6–10)
Urgency	
Never, rarely	6 (75%)
Sometimes	2 (25%)
Incontinence	
Never, rarely	6 (75%)
Sometimes	2 (25%)
Seepage	
Daytime	5 (63%)
Nighttime	4 (50%)
Pad use	
Daytime	4 (50%)
Nighttime	5 (63%)
Reason for pad use (*n* = 7)	
Necessity	3 (43%)
Peace of mind	4 (57%)
Restrictions	
Dietary	1 (13%)
Social	1 (13%)
Work	1 (13%)
Sexual	1 (13%)
CGQL (*n* = 53)	8.3 (8–10)
Recommendation of surgery to others	8 (100%)
Willingness to undergo surgery again if needed	8 (100%)

^a^ Several patients had several complications.

## 4 DISCUSSION AND CONCLUSIONS

We present our experience and outcomes of patients undergoing revisional/redo IPAA surgery who were initiated on biologic therapy. Beyond an initial case report, this is the first study where combined biological and surgical therapy used in the setting of CD‐like features.^6^ On short‐term follow‐up, our series revealed a functioning pouch rate of 75% after revisional/redo IPAA surgery. While 71% of patients underwent revisional/redo IPAA with coverage of biologics, 29% of patients re‐initiated biologics after second stage of redo IPAA due to the new CD‐like features such as the presence of a perianal fistula.

Pouch retention rates vary in patients undergoing revisional/redo IPAA surgery. A previous report from the senior author (FR) examining 30 patients with CD who underwent redo IPAA reported a 5‐year pouch survival rate of 55% over a median follow‐up period of 2.2 years.^8^ Garrett et al. evaluated 33 patients with CD of the pouch who subsequently underwent redo pouch surgery and estimated a pouch retention rate of 85% with a median follow‐up of 1.7 years.^9^ Notably, only seven patients (21.2%) had pathologic findings in the pouch specimens suggestive of CD.

Half of our patients presented with fistulas, primarily originating from the previous IPAA anastomosis. Heimann et al. similarly reported that patients who developed perianal fistulas after ileoanal pouch surgery were significantly more likely to have partial dehiscence or stricture of the ileoanal anastomosis. Additionally, they found that patients with IC and CD were more prone to developing perianal fistulas compared to those with UC (16% vs. 3%, *p* < 0.001).^10^ Furthermore, in a large cohort of 1965 patients who developed pouch‐related fistulas after IPAA surgery, individuals with CD or IC were 2.9 and 1.8 times more likely to develop pouch‐related fistulas, respectively.^11^ Patients included in our study showed similar rates of perianal fistulas and pouch‐vaginal fistulas, with 17% of patients having previously undergone seton placement. Treating CD‐related pouch‐vaginal fistulas poses significant challenges, and there are various surgical options available. In a retrospective analysis of 102 patients with pouch‐vaginal fistulas, 75 patients underwent local repair, including the use of advancement flaps in 48 cases and transvaginal repair in 27 cases. Healing of the fistula was significantly lower, and pouch failure was significantly higher in CD‐related pouch‐vaginal fistulas compared to non‐CD‐related pouch‐vaginal fistulas.^12^

In our study, 17 patients (71%) of patients also experienced at least one mechanical issue, with nine patients (38%) having a long rectal cuff as observed during pouchoscopy. A long rectal cuff increases the risk of kinking and obstruction, leading to pouch failure.^13^, ^14^ Therefore, it is crucial to check the position of the pouch before creating the anastomosis. If feasible, especially in the presence of a long rectal cuff, a stapled redo IPAA anastomosis is the preferred approach. However, if no retained rectum is present, a mucosectomy with a handsewn anastomosis becomes the option. In our study, all of the redo pouches were created as J pouch configurations, and the majority of the anastomoses were performed using a handsewn technique with mucosectomy, which is our preferred technique in the setting of concern for CD. Mucosectomy and hand‐sewn technique eliminates the potential source for perianal inflammation when there is a concern for CD.

To the best of our knowledge, our study represents the first investigation of continued use of biologics during redo IPAA surgery. There is currently no published data supporting routine postoperative prophylaxis with advanced therapies to prevent the development of CD of the pouch, and this approach is not recommended for patients with IC.^7^ This observational study, conducted over a 6‐year period at a high‐referral center, focuses on salvage strategies for redo pouch surgery. While it does not aim to provide definitive conclusions regarding the diagnosis of CD, it highlights the potential for subjective clinical interpretations to evolve into objective findings in future studies building on this pilot work. In these settings, once again, our study emphasizes the importance of complementing biologics in the setting of reoperative pouch surgery when patients have severe aversion to permanent ileostomy.

We hope that integrating biologic therapy as an adjunct to redo pouch surgery can open future frontiers and possibilities, particularly for patients with a strong aversion to permanent ileostomy. This is especially relevant in cases where a pathological diagnosis of CD cannot be confirmed, but clinical and radiological findings suggest its possibility. In such scenarios, biologic therapy could offer additional options to avoid a permanent stoma, providing hope and alternative strategies for this challenging patient population and should at the very least be considered.

While our experience and findings are novel, our study does have several limitations. First, we included a limited number of patients treated at a single referral‐based institution, and the follow‐up time varied among the patients. In addition, a proportion of patients had their initial IPAA surgery performed at another center, and many of the externally referred patients had already been prescribed advanced therapies, which introduces potential confounding factors. Furthermore, our study lacks a comparator group of patients undergoing revisional/redo IPAA surgery who were not treated with biologics or small molecules. Lastly, we did not thoroughly evaluate the efficacy and complications of advanced therapies and primarily focused on surgical treatment outcomes.

In conclusion, this study serves as an initial report of a promising concept, suggesting that revisional/redo IPAA surgery may be a successful approach for managing pouch failure in selected patients with CD‐like features of the pouch. We recognize that further studies are necessary to definitively evaluate the impact of biologics on pouch outcomes in this setting. Therefore, this initial study lays the groundwork and establishes a framework for future prospective research in this field.

## AUTHOR CONTRIBUTIONS

**Mehmet Gulmez:** Conceptualization; formal analysis; methodology; writing – original draft; writing – review and editing. **Daniel J. Wong:** Conceptualization; methodology; writing – review and editing. **Ariela K. Holmer:** Conceptualization; resources; writing – review and editing. **Eren Esen:** Conceptualization; data curation; methodology; validation. **Shannon Chang:** Conceptualization; investigation; visualization; writing – review and editing. **Arman Erkan:** Conceptualization; investigation; resources. **David Hudesman:** Conceptualization; project administration; supervision. **Andre da Luz Moreira:** Writing – review and editing. **Feza H. Remzi:** Conceptualization; investigation; methodology; project administration; resources; supervision; writing – review and editing.

## FUNDING INFORMATION

The authors received no financial support for the research, authorship, and/or publication of this article.

## CONFLICT OF INTEREST STATEMENT

Dr. Feza Remzi is an Editorial Board Member of *AGS*. The authors declare that they have no other conflicts of interest relevant to this manuscript.

## ETHICS STATEMENT

Approval of the research protocol: This study was approved by the Ethics Committee of NYU Langone Health (i23‐01115).

Informed Consent: Due to the retrospective design of this study, the requirement for informed consent was waived.

Registry and the registration no. of the study/trial: N/A.

Animal studies: N/A.

## Data Availability

The authors will make study raw data available to other researchers upon request from the corresponding author.
